# A critical base pair in k-turns determines the conformational class adopted, and correlates with biological function

**DOI:** 10.1093/nar/gkw201

**Published:** 2016-03-25

**Authors:** Lin Huang, Jia Wang, David M. J. Lilley

**Affiliations:** Cancer Research UK Nucleic Acid Structure Research Group, MSI/WTB Complex, The University of Dundee, Dow Street, Dundee DD1 5EH, UK

## Abstract

k-turns are commonly-occurring motifs that introduce sharp kinks into duplex RNA, thereby facilitating tertiary contacts. Both the folding and conformation of k-turns are determined by their local sequence. k-turns fall into two conformational classes, called N3 and N1, that differ in the pattern of hydrogen bonding in the core. We show here that this is determined by the basepair adjacent to the critical G•A pairs. We determined crystal structures of a series of Kt-7 variants in which this 3b,3n position has been systematically varied, showing that this leads to a switch in the conformation. We have previously shown that the 3b,3n position also determines the folding characteristics of the k-turn, i.e. whether or not the k-turn can fold in the presence of metal ions alone. We have analyzed the distribution of 3b,3n sequences from four classes of k-turns from ribosomes, riboswitches and U4 snRNA, finding a strong conservation of properties for a given k-turn type. We thus demonstrate a strong association between biological function, 3b,3n sequence and k-turn folding and conformation. This has strong predictive power, and can be applied to the modeling of large RNA architectures.

## INTRODUCTION

Large RNA species have complex structures, with extensive secondary and tertiary interactions. However, the secondary structure can be simplified by considering it to comprise a series of approximately rigid duplex segments connected by helical junctions. The relative trajectories of the helices are determined by the junctions, so mediating long-range tertiary interactions. To a first approximation it is the junctions that set up the overall architecture of the molecule, and it is therefore important to understand their conformational and folding properties.

**Figure 1.**
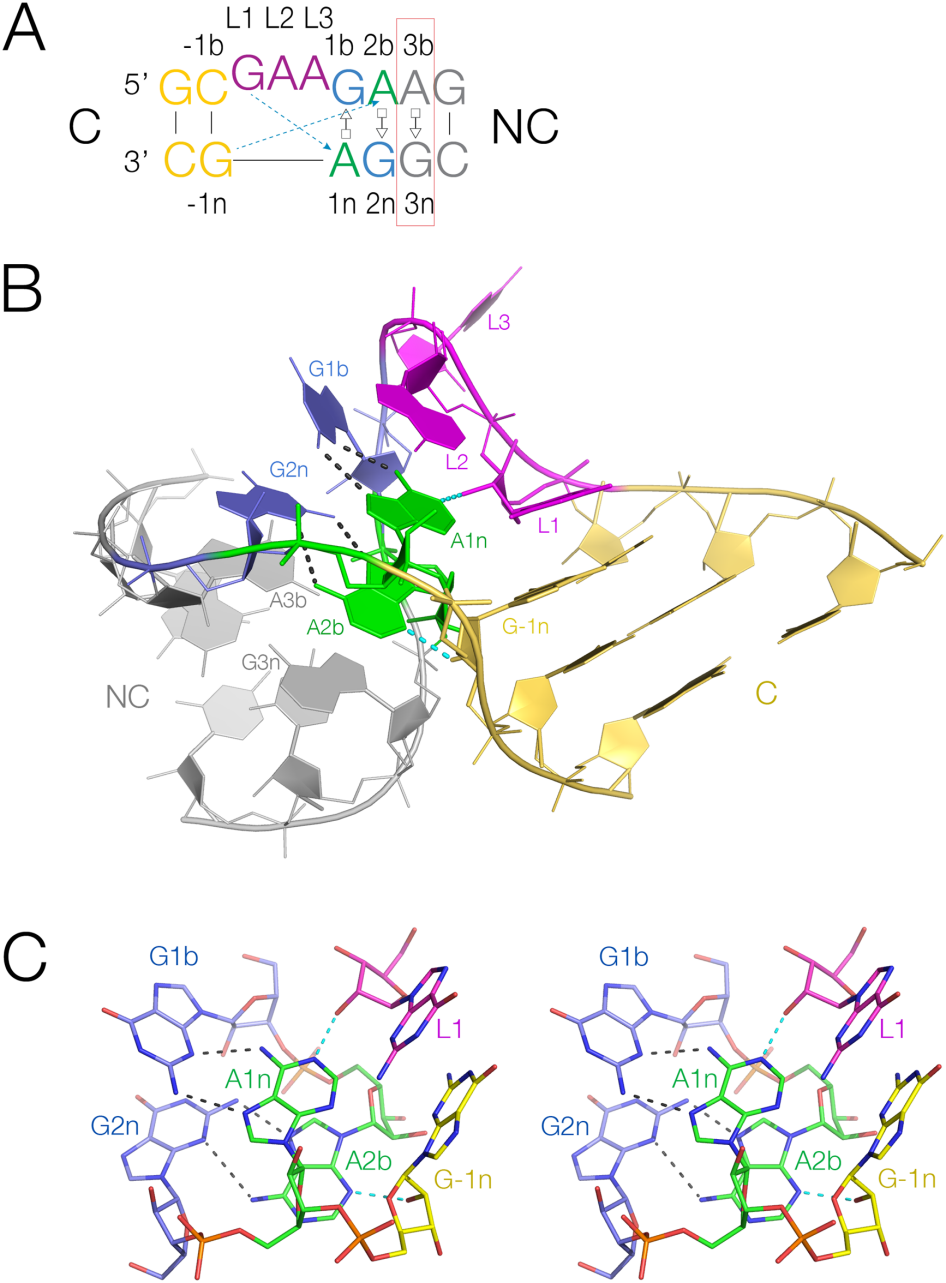
Standard k-turn sequence and structure. (**A**) The sequence of *Haloarcular marismortui* Kt-7, with the nucleotide positions labeled using the established nomenclature (1). The 3b,3n basepair studied here is boxed red. The two key cross-strand hydrogen bonds are shown as broken arrows colored cyan. (**B**) The structure of HmKt-7. The coloring matches that of the sequence in part A. The bulged strand is at the back in this view, with the non-canonical helix (NC) on the left, and the canonical helix (C) on the right. (**C**) The A-minor hydrogen bonds (broken lines) in the core of the k-turn structure shown in parallel-eye stereoscopic view. The two cross-strand hydrogen bonds are highlighted cyan.

One especially common junction element is the kink-turn (k-turn) (Figure 1). k-turns are elements in double-stranded RNA that introduce a tight kink into the helical axis, with an included angle close to 50°. A standard k-turn comprises a three-nucleotide bulge followed by successive G•A and A•G *trans* G(sugar edge)•A(Hoogsteen edge) basepairs. The standard nomenclature of nucleotide positions (1) is shown in Figure 1A. One of the component helices is often a relatively short stem-loop that interacts with a receptor at a remote location in the RNA. Thus, an important function of k-turns is to mediate tertiary interactions, and the majority of the k-turns in the ribosome make such contacts for example. In addition, most k-turns bind specific proteins which may help to stabilize the kinked structure. In general k-turns may exist in an extended structure or the more characteristic kinked structure. Some, but not all, k-turn sequences fold in response to addition of metal ions (1,2). k-turns may also be induced to fold by formation of tertiary contacts (3) or by the binding of proteins including members of the L7Ae family (4–7).

The k-turn folds by juxtaposition of the minor grooves of the helices on either side of the bulge (Figure 1B). A series of hydrogen bonds are formed at the interface, with particularly critical cross-strand bonds accepted by the adenine bases of the G•A and A•G pairs (1,8–10) (Figure 1C, also indicated by broken cyan arrows in part A). One is donated by the loop L1 O2’ and accepted by A1n N1, i.e. the conserved adenine in the G•A pair closest to the bulge. The O2’ of the ribose in the -1n position (in the first basepair of the helix 5′ to the bulge) donates the second key hydrogen bond, that is accepted by a ring nitrogen atom of the conserved adenine at the 2b position.

However, analysis of the structures of all available k-turns revealed that they naturally divide into two classes, depending on the acceptor of the hydrogen bond donated by the -1n O2' (10). In approximately half the cases the acceptor is A2b N3, while in the other half it is A2b N1. This requires a significant reorientation of the A2b nucleobase, so that while the basepair with G2n is connected by two hydrogen bonds in the N3-bonded structure, in the N1 structure the distance between A2b N6 and G2n N3 is typically longer than 4 Å, i.e. not hydrogen bonded. This is associated with a rotation of the axis of the C helix (10), which could potentially affect the formation of tertiary contacts mediated by the k-turn.

The question therefore arises as to what determines whether a given k-turn adopts the N3 or the N1 structure. Given the conservation of many elements of the structure, especially the G•A pairs, the possibilities are rather limited. We have recently shown that the 3b,3n sequence (i.e. the next base pair after the G•A pairs moving away from the loop in the NC helix) determines whether a k-turn will fold in response to the addition of metal ions alone (11). We therefore considered the possibility that the 3b,3n sequence might also affect the conformation adopted, and find that this is indeed the case. The goal of these studies is to generate a set of rules allowing us to predict the folding and conformational properties of k-turns in unknown RNA structures.

## MATERIALS AND METHODS

Full experimental details are present in Supplementary Information.

### RNA synthesis

Ribooligonucleotides were synthesized, deprotected and purified as described in Wilson *et al*. (12).

### Preparation of SAM-I riboswitch variants

This RNA was generated using a plasmid containing a gene encoding the *Thermoanaerobacter tengcongensis* SAM-I riboswitch (13) in which the natural k-turn was replaced by HmKt-7 (10). Substitutions at the 3b,3n position were generated by site-directed mutagenesis. The SAM-I riboswitch variants were transcribed from PCR-amplified templates using T7-RNA polymerase.

### Expression and purification of human U1 snRNP protein A and *A. fulgidus* L7Ae

U1A-RBD (residues 1–102) (14) was expressed in the *Escherichia coli* BL21-Gold (DE3) pLysS cells (Stratagene) using a T7 RNA polymerase expression vector. An extract of lysed cells was purified by chromatography on CM columns (GE Healthcare), a heparin column (GE Healthcare) and finally by gel filtration. The gene encoding full-length *A. fulgidus* L7Ae was cloned into a modified pET-Duet1 plasmid (Novagen) (15). A hexahistidine-L7Ae fusion protein was expressed in *E. coli* BL21-Gold (DE3) pLysS cells (Stratagene). The protein was purified by chromatography on HisTrap (GE Healthcare). After removal of the six-histidine tag it was further purified by chromatography on heparin followed by gel filtration on Superdex 200.

### Crystallization, structure determination and refinement

#### Kt-7 determined in different structural environments

Two types of design were used to crystallize Kt-7 RNA in the absence of protein (Supplementary Figure S1). (i) a 24 nt RNA with a GAAA tetraloop closing the C-helix, and (ii) a 19 nt RNA oligonucleotide with two inverted copies of the Kt-7. Solutions of 1 mM RNA in 5 mM Tris-HCl (pH 8.0), 100 mM NaCl were heated to 95°C for 1 min, and slowly cooled to 20°C. MgCl_2_ was then added to a final concentration of 10 mM. Crystallization was performed using the hanging-drop vapor diffusion method.

#### Stem loop (a) - corresponding to PDB 5FJ1

Crystals of space group P2_1_2_1_2_1_ were obtained and a 2.75 Å resolution data set was collected on beamline I03 of the Diamond Light Source. The structure was determined by molecular replacement using the program PHASER (16) with *H. marismortui* Kt-7 (PDB 4BW0) as the search model.

#### Simple duplex (b) - corresponding to PDB 5FJ0

Crystals of space group P4_2_22 were obtained. A 2.2 Å resolution data set was collected on beamline ID29 at the European Synchrotron Radiation Facility (ESRF). The structure was determined by molecular replacement using the program PHASER (16) with *H. marismortui* Kt-7 (PDB 4CS1) as the search model.

#### Kt-7 bound to L7Ae and U1A - corresponding to PDB 5FJ4

A total of 0.25 mM RNA was mixed with 0.25 mM U1A-RBD and 0.25 mM L7Ae in 5 mM Tris-HCl (pH 8.0), 100 mM NaCl, 10 mM MgCl_2_ and crystals were by vapor diffusion. Crystals of space group C222_1_ were determined by molecular replacement using the program PHASER (16) with PDB 4C4W as the search model.

#### The SAM-I riboswitch Kt-7 variants

The SAM-I riboswitch variants were crystallized using the hanging drop method. Drops were seeded using a micro-crystals taken from crystal trays containing the unmodified RNA (corresponding to structure PDB 4B5R). Crystals of space group P4_3_2_1_2 were obtained. Diffraction data were collected on different beamlines, including I02, I03, I04, I04-1 and I24 at Diamond Light Sources and ID23-1 and ID29 at ESRF. Data were indexed, integrated and scaled using XDS (17) or iMOSFLM and Scala from the CCP4 suite of programs (18,19). Structures were solved by performing molecular replacement using PDB entry 3GX5 (13) or 4B5R as a preliminary model. The structures were refined using Phenix refine, and the model was built using COOT (20). The composite omit map was calculated using Phenix (21).

### Sequence alignment and analysis

Bacterial Kt-7 and Kt-46 sequences were taken from the Comparative RNA website. Specific k-turn regions were aligned manually using Jalview 2.8 (22). This resulted in the analysis 2722 of Kt-7 and 3181 of Kt-46 sequences. A total of 4755 SAM-I riboswitch and 9235 U4 snRNA sequences were taken from the Rfam database (23). All sequence composition and covariation analysis was calculated using a modified version of Jalview, that was kindly provided by Dr James Procter (University of Dundee).

## RESULTS

### The two classes of k-turn conformation

**Figure 2.**
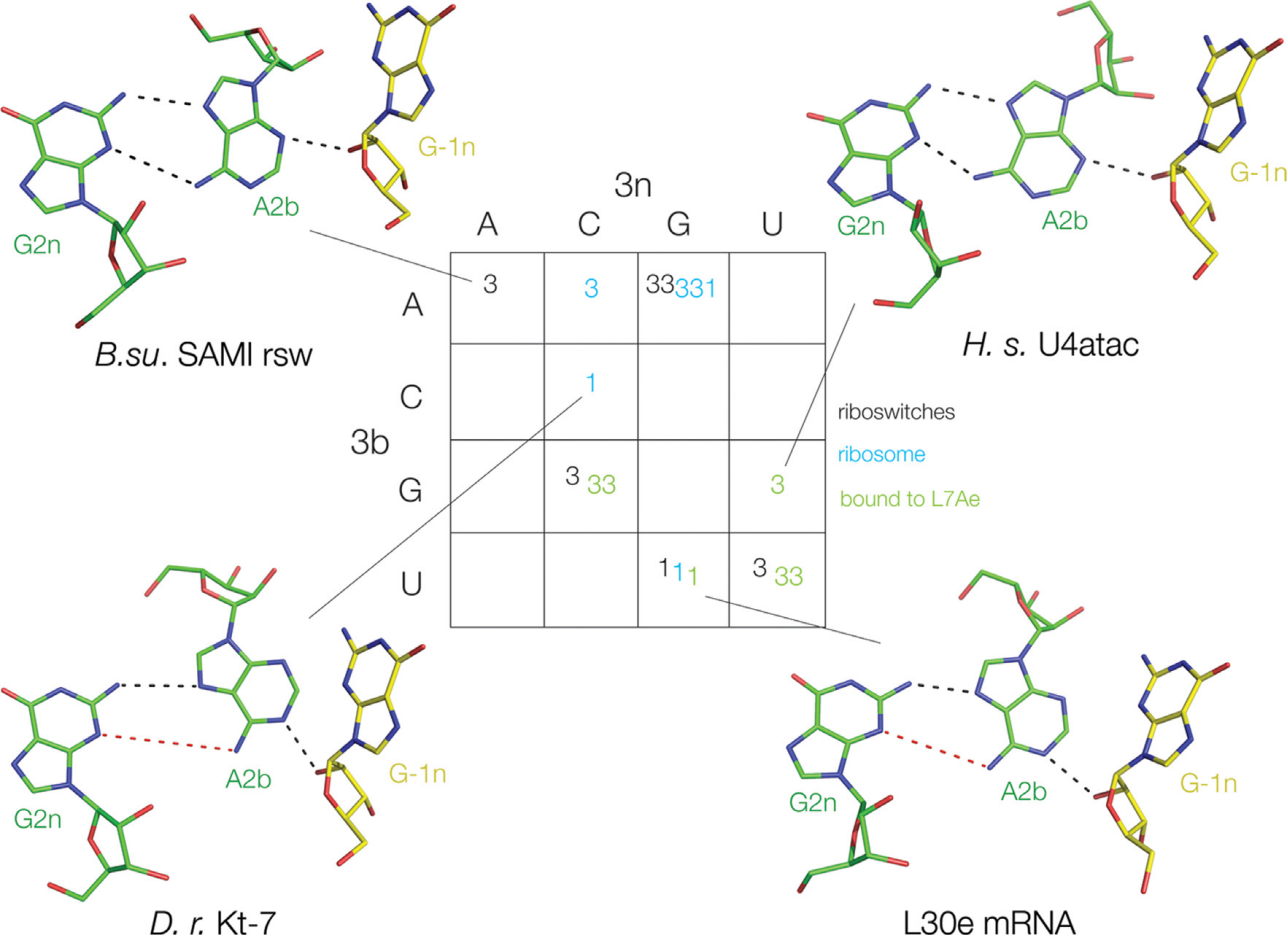
The naturally-occurring k-turns of known structure classified as N3 or N1 conformations as a function of their 3b,3n sequences. In this and subsequent figures, the k-turns are placed within the cells of a 4 × 4 array according to their 3b (*rows*) and 3n (*columns*) sequences. Here, the conformation of each k-turn is shown as N3 (*3*) or N1 (*1*) conformation. The origin of each k-turn is shown as riboswitch (black), ribosome (cyan) or as an L7Ae complex (green). The structures of the 2b•2n•-1n triple base interactions are shown for four of these k-turns. Hydrogen bonds are indicated by black broken lines. Red broken lines indicate distances too long to be hydrogen bonded.

The structures of 18 natural k-turns have been determined in different environments (Supplementary Table S1), including those located in riboswitches and ribosomes, and bound by different proteins such as L7Ae. Of these, 13 are in the N3 conformation while the remaining 5 are in the N1 conformation. The defining difference lies in one of the key cross-strand hydrogen bonds connecting the minor groove edges of the C and NC helices, specifically the receptor for the proton donated by the O2' of -1n, i.e. A2b N3 or N1; examples of each class are presented in Figure 2. There appears to be no particular correlation with the type of environment. For example, the k-turns of the SAM-I and cobalamine riboswitches are N3 and N1 conformation, respectively, and both N3 and N1 are found in ribosomal k-turns. We therefore examined the sequence environment of the different k-turns.

### A correlation between the structural class of natural k-turns and the 3b•3n sequence

The standard k-turns all have G•A and A•G pairs at the 1b,1n and 2b,2n positions, and generally a Watson–Crick C–G pair at the -1b,-1n position, together with a 3-nucleotide loop of various composition. Any sequence determinant of structure is unlikely to reside within these elements. We have previously found that the sequence at the 3b,3n position is important in determining the folding characteristics of k-turns, i.e. whether or not they can be induced to fold into the tightly-kinked structure by addition of metal ions alone. We therefore considered the possibility that the 3b,3n sequence might also influence the conformation adopted in the folded form of the k-turn.

A full tabulation of all the structures analyzed in this study is given in Supplementary Table S1, and the 3b,3n sequences are presented in array form in Figure 2. In this 4 × 4 array, the rows are the 3b sequences and the columns the 3n sequences. The N3 or N1 conformation adopted by each k-turn is written into a cell of the array according to its 3b,3n sequence. The structures of the G2n•A2b•A-1n triples are shown for four examples, two each of N3 and N1 structures. The k-turns in this array encompass 8 of the 16 possible 3b,3n sequences; in four cases a given 3b,3n sequence is found in several different k-turns. These are particularly interesting, because they reveal that with one exception, a given 3b,3n sequence is associated with a single conformation. Thus, there are three examples each of 3b,3n = G–C and U•U, all of which are k-turns that adopt the N3 conformation. There are also three examples of 3b,3n = U•G; in this case each adopts the N1 conformation. Interestingly, each 3b,3n = U•G k-turn exists in a different environment, in a riboswitch (cobalamine (24)), ribosome (*Thermus thermophilus* Kt-7 (25)), or bound by the ScL30e protein (26). Yet the correlation between 3b,3n = U•G and the N1 conformation holds good for each.

### Ribosomal Kt-7 is the exception

The correlation between 3b,3n sequence and conformation is strong, suggesting that this might be used predictively. However, there is one exception to the apparent generality that a given 3b,3n sequence is associated with a single conformation. The class 3b,3n = A•G is the largest, with five members; there is a probable reason for this, that we shall discuss below. Four of these are N3 structures (the SAM-I riboswitch k-turn with and without bound YbxF protein (27), and the ribosomal k-turns Kt-46 and Kt-78), but the fifth is N1. The exceptional N1 structure is Kt-7 observed in the *Haloarcula marismortui* ribosomal 50S subunit (28). However, we have shown previously that in other environments, this k-turn (abbreviated here to HmKt-7) adopts the N3 structure (10). In the course of our studies of k-turns we have determined numerous structures of HmKt-7 removed from the ribosomal context including three new structures reported here, all of which are N3 structures (Supplementary Table S2). These include a stem loop (this work PDB 5FJ1; Supplementary Figure S1A), simple duplexes (PDB 4C40, 4CS1) (11) plus a new structure determined here (PDB 5FJ0; Supplementary Figure S1B), as RNA bound by L7Ae protein (PDB 4BW0) (7) or by L7Ae and U1A (this work PDB 5FJ4; Supplementary Figure S1C) and in the SAM-I riboswitch (PDB 4B5R) (10). Moreover, the various structures, with resolutions in the range 1.7 to 3.1 Å, have been determined in five different space groups, encompassing hexagonal, tetragonal and orthorhombic symmetries. Several include multiple molecules within the asymmetric unit and altogether there are 17 independent structures, all of which are unambiguously in the N3 conformation, and all in a closely similar conformation with a small RMSD values (Supplementary Table S3). Thus it is clear that the N1 conformation adopted by HmKt-7 in the ribosome is the single exception. In the ribosome the k-turn mediates an important tertiary interaction, and is bound by the L24 protein (28) (Supplementary Figure S2), and it is likely that these factors force the conformation against its intrinsic nature. Moreover, in the large majority of Kt-7 sequences 3b,3n is not A•G, but rather N1-preferring sequences (see below).

### The influence of 3b•3n sequence on Kt-7 conformation

The correlations observed above clearly raise the possibility of a causative relationship between 3b,3n and k-turn conformation. We therefore decided to extend the study with a systematic structural analysis of HmKt-7 as a function of the 3b,3n sequence. This was performed in the constant environment of the SAM-I riboswitch, modified by replacing its natural k-turn by HmKt-7 with different 3b,3n sequences (Supplementary Figure S3). In this way we could observe the effect of changing the 3b,3n sequence alone in an otherwise completely unchanged RNA molecule. Additionally, we hoped we might expand the range of 3b,3n sequences beyond the eight found in the natural k-turns to date.

**Figure 3.**
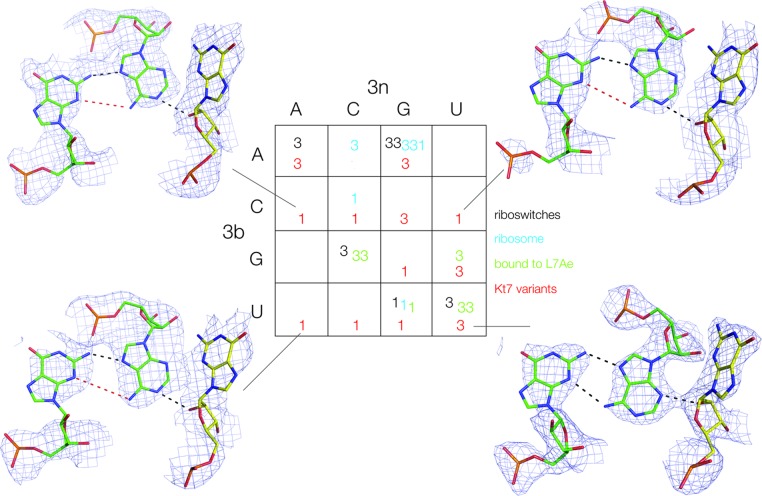
The conformation of HmKt-7 3b,3n-variants within the SAM-I riboswitch context. The structures of 12/16 variants have been determined from which we have deduced the N3 (*3*) or N1 (*1*) conformation (see also Supplementary Figure S3). These are shown in red within the 4 × 4 array, added to those of the natural k-turns colored as in Figure 2. The structures of the 2b•2n•-1n triple base interactions are shown for four of the modified Kt-7 k-turns. Hydrogen bonds are indicated by black broken lines. Red broken lines indicate distances too long to be hydrogen bonded. The complete set of 2b•2n•-1n triple base structures is presented in Supplementary Figure S4.

Altogether we obtained structures at better than 3.3 Å resolution for SAM-I riboswitch containing HmKt7 variants with twelve of the possible 3b,3n sequences (Supplementary Table S4). Examples of these structures are shown in Figure 3 and the complete set in Supplementary Figure S4. At this resolution the assignment of N3 or N1 structures is unambiguous, and the results are shown as a function of 3b,3n sequence in array form in Figure 3, with the new Kt-7 variant data highlighted in red. Six are completely new, i.e. 3b,3n = C•A, C–G, C•U, G•G, U–A and U•C, five of which adopt the N1 conformation. Although we have not been able to obtain structures with all possible 3b,3n sequences, 14 of the 16 possible cells of the array now contain data, leaving only two unknown. Where the modified HmKt-7 elements have the same 3b,3n sequence as a different k-turn, they adopt the same conformation as that structure. Altogether seven of the new structures are in the N1 conformation, and clearly simply changing the 3b,3n sequence alone is sufficient to induce the structure of Kt-7 to change within the riboswitch environment. Evidently the 3b,3n sequence is the major determinant of the k-turn conformation.

### The influence of the 3b,3n sequence on folding and conformation of the k-turn

**Figure 4.**
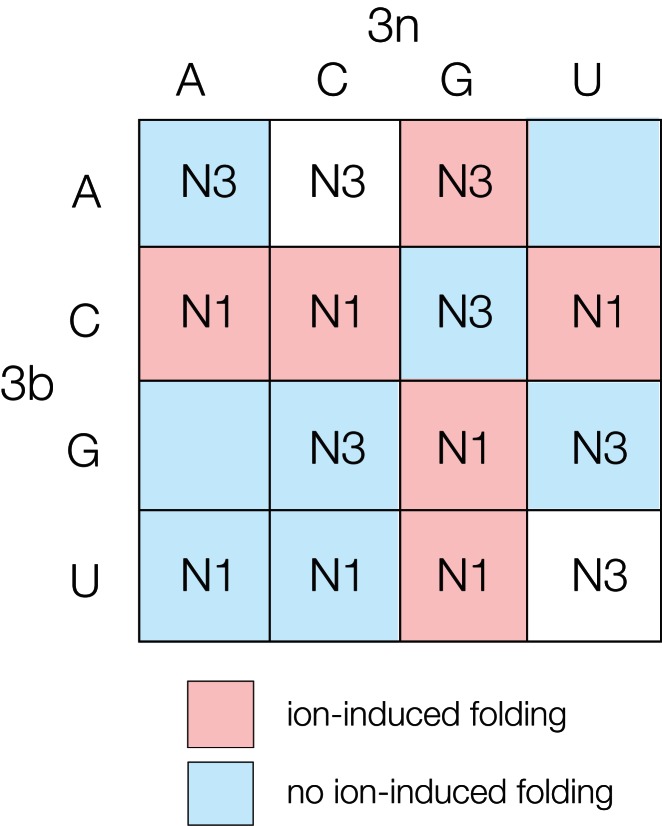
Summary of the conformation and folding characteristics of k-turns according to their 3b,3n sequences. Each cell of the array is labeled with the N3 or N1 conformation associated with that 3b,3n sequence where determined. The cells are colored by the ability of those k-turns to undergo ion-induced folding (11). k-turns that undergo folding on addition of metal ions alone are colored red, while those that do not are colored blue.

The 3b,3n sequence clearly influences both the conformation of the k-turn, i.e. whether it adopts the N3 or N1 structure, and its ability to be folded by metal ions alone (11). Both sets of data are summarized by the array shown in Figure 4. This shows the conformation associated with each 3b,3n sequence, while each cell is colored by the ability of the k-turn to undergo ion-induced folding (put simply, red can be folded, blue cannot) (11). The main rules for the ion dependence of folding are that k-turns in which 3b = C (row 2) and 3n = G (column 3) fold in metal ions, 3b,3n = Watson–Crick basepair (ascending diagonal) are not folded by ions and that the latter dominates because 3b,3n = C–G is not folded in metal ions alone. While the connection between 3b,3n sequence and conformation is strong, no such simple pattern can be extracted, and any generalizations have exceptions. There is a tendency for 3b = purine to result in an N3 structure, but the generality is broken by the N1 structure adopted by the 3b,3n = G•G Kt-7 variant. We note the majority (6/8) of the 3b = pyrimidine k-turns form N1 structures. 3b,3n = A•G is the only k-turn that folds in response to addition of metal ions to adopt the N3 conformation. While we cannot draw up a set of simple rules relating 3b,3n sequence and k-turn conformation, the array can be used as a look-up table in order to predict the properties of a new k-turn of unknown folding and conformation.

We have analyzed the structures of the basepairs formed by the 3b,3n pairs in 30 k-turn structures, i.e. the 18 natural k-turns plus 12 Kt-7 variants determined here (Supplementary Figure S5). We find that in the seven cases where there are distinct k-turns with a common 3b,3n sequence, all have the same kind of 3b,3n basepair (for classification of basepairing see Leontis and Westhof (29)). For example, the 6 structures with 3b,3n = A•G all form *trans*-Hoogsteen-sugar edge basepairs. Interestingly, over the whole set of data we only observe two kinds of basepair formed by 3b,3n (Supplementary Tables S1 and S4). They are either *trans*-Hoogsteen-sugar edge or *cis*-Watson–Crick pairs. However, there is no clear correlation between the type of 3b,3n basepair and the adoption of N3 or N1 structure in that both N3 and N1 k-turns divide equally between the two types of basepair.

**Figure 5.**
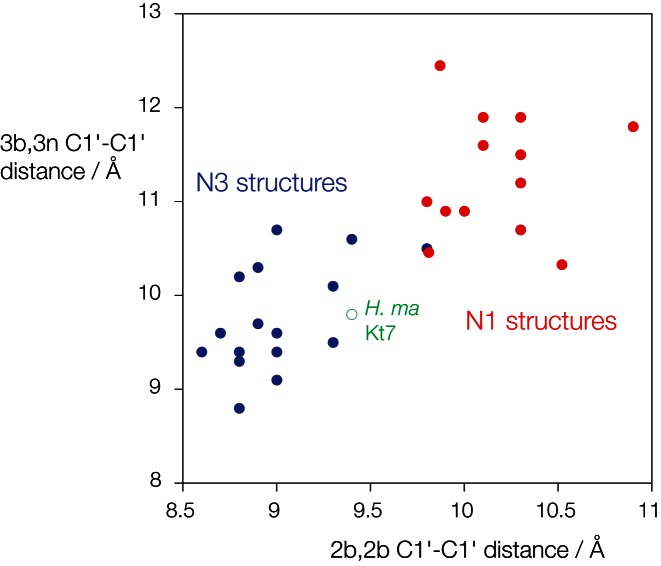
Correlation of C1'–C1' distances for the 2b•2n and 3b•3n basepairs of known k-turn structures. The two C1'–C1' have been measured for each k-turn structure and plotted as shown. N3 structures are plotted in blue, N1 structures in red. Note that the two groups form two distinct clusters of points such that the N3 structures evidently are associated with a narrower NC helix. The exceptional HmKt-7 structure within the ribosome is plotted in green using an open circle. An additional plot identifying each k-turn and showing its 3b,3n sequence is shown in Supplementary Figure S6.

The structural switch between the N3 and N1 conformations requires a rotation of the A2b nucleobase, and this leads to a change in the local helical width. Analyzing all the available structural data, the C1'-C1' distances in the 2b•2n basepairs for the N3 structures have a mean value of 8.93 ± 0.22 Å, while those for the N1 structures have a mean value of 10.17 ± 0.40 Å. It seemed very possible that the width of the 3b•3n basepair might affect the stability of the structure, thus influencing the relative stability of the two conformations. We therefore plotted the C1'-C1' distances for the 2b•2n and 3b•3n basepairs for our complete set of k-turns to see if a correlation existed (Figure 5). In fact, the two distances are strongly positively correlated, and cluster into two distinct groups corresponding to the N3 and N1 structures (plotted blue and red respectively). The mean C1'-C1' distances for the 3b•3n basepairs are 9.59 ± 0.65 Å and 11.30 ± 0.65 Å for the N3 and N1 structures respectively. The N3 group contain a preponderance of 3b,3n = A•G and U•U pairs, and the N1 group comprise mainly 3b,3n = U•G, C•A and C•C pairs (Supplementary Figure S6). This is fully consistent with the array analysis above.

### Bioinformatic analysis of the correlation between 3b•3n sequence and function

**Figure 6.**
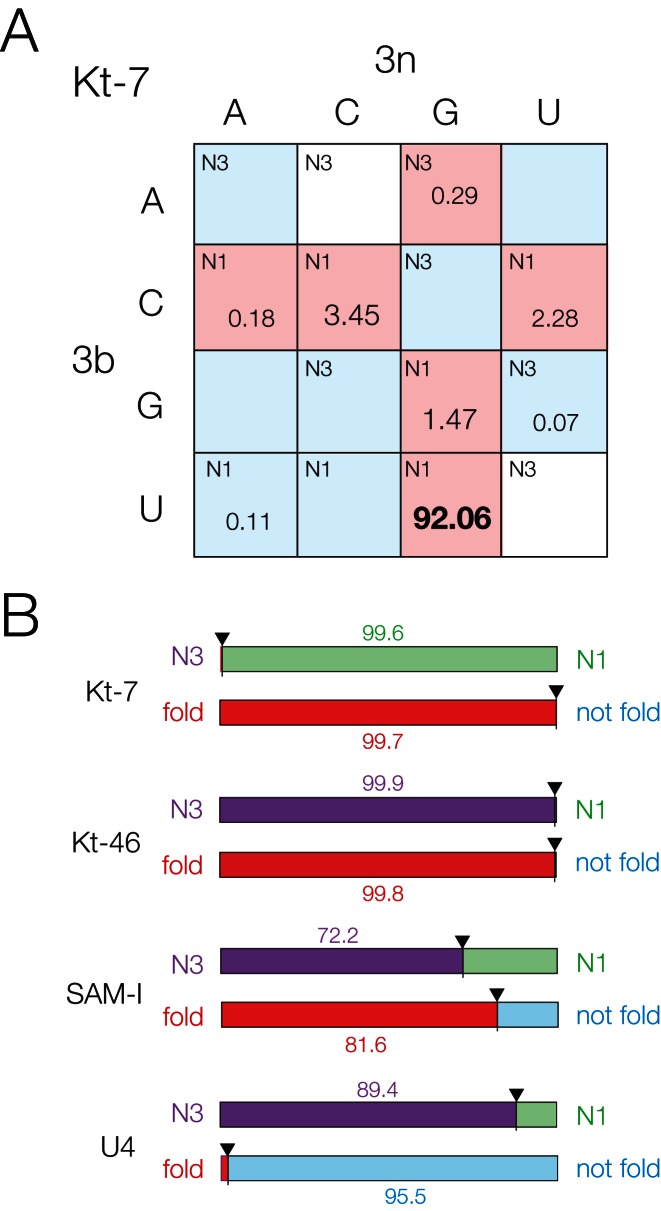
Bioinformatic analysis of four natural k-turns according to their 3b,3n sequences. (**A**) The distribution of 2722 natural k-turn sequences of bacterial Kt-7 according to the 3b,3n sequences. Each cell is indicated by its preferred conformation (*N3* or *N1*), and colored by its ion-induced folding properties. The fraction of Kt-7 sequences (as a percentage of the total) for each 3b,3n sequence is indicated for non-zero values. The largest fraction (3b,3n = U•G, 92.06%) is highlighted bold. The 4 × 4 arrays are shown for all four k-turns analyzed in Supplementary Figure S7. (**B**) Bar plots summarizing the deduced conformation and folding preferences for bacterial Kt-7, Kt-46, SAM-I riboswitch and U4 snRNA k-turns. For each k-turn type two plots are shown. The upper one indicates the probable fractional population of N3 (purple) versus N1 (green) conformation, while the lower plot indicates the likely ion-induced folding characteristics between folding (red) and non-folding (blue) in metal ions. These properties are deduced on the basis of the application of the rules indicated by Figure 4. In each case the bars are labeled with the percentage of the larger fraction.

With the above properties in mind it is informative to look at the distribution of 3b,3n sequences in various k-turns of RNA species of different functions, using thousands of k-turn sequences taken from the Comparative RNA website and the RFAM database (23). Arrays showing the distribution of 3b,3n sequences for four different k-turns are shown in Supplementary Figure S7. The predicted conformational and folding properties for these k-turns are summarized as bar charts in Figure 6, plotting the fraction of species with either predicted N3 (purple) versus N1 (green) conformation (upper bar in each case), and possessing (red) or lacking (blue) the predicted ability to undergo ion-induced folding (lower bar).

First, we compare two contrasting ribosomal k-turns. Analysis of 2722 bacterial Kt-7 sequences shows that 99.4% of these have 3b,3n sequences that confer the N1 conformation. The majority of these (totaling to 99.7%) conform to either 3b = C or 3n = G, conferring ion-induced folding (Figure 6A). A marked preference (92.06%) for 3b,3n = U•G is clear. This shows that bacterial Kt-7 sequences are expected to be overwhelmingly biased toward the N1 conformation together with an ability to fold in metal ions alone. We noted above that the Kt-7 of *H. marismortui* seems to be an exceptional case with 3b,3n = A•G. As we have seen this is forced to adopt an N1 conformation in the ribosome in spite of the 3b,3n sequence, and within the bacterial Kt-7 sequences 3b,3n = A•G represents a very small minority (0.29%).

Kt-46 is an N3-conformation k-turn with an extremely strong sequence bias, adopting a different conformation from Kt-7. Analysis of 3181 Kt-46 sequences from bacterial ribosomes reveals that at least 99.94% have 3b,3n sequences that confer the N3 conformation, and 99.75% are A•G (Supplementary Figures 6B and S7). As noted above, 3b,3n = A•G is the only sequence that confers both an N3 conformation and ion-induced folding, suggesting that these properties are essential for Kt-46 in the context of bacterial ribosome formation.

The structure of the *Thermoanaerobacter tengcongensis* SAM-I riboswitch (13) revealed that it contained a structurally-important k-turn, whose sequence is quite similar to that of the *H. marismortui* Kt-7 including 3b,3n = A•G. Analysis of 4755 SAM-I riboswitch k-turn sequences reveals a less-tightly distributed set of inferred folding properties (Figure 6B, Supplementary Figure S7). Although 73.4% of the sequences should confer an N3 conformation, a significant minority (21.7%) are likely to adopt the N1 structure. This is consistent with our finding that both N3 and N1 structures can be accommodated within the fold of the SAM-I riboswitch. Overall, 81.7% of the SAM-I k-turns fit the 3b = C or 3n = G requirement for ion-induced folding.

Lastly, we have analyzed the distribution of 3b,3n sequences in 9235 U4 snRNA k-turns (Figure 6B, Supplementary Figure S7). Unlike the other k-turns analyzed, the U4 data occupy every cell in the 3b,3n array. Nevertheless, clear bias can be found. At least 89.4% of the sequences are predicted to be N3-forming. Moreover 7.1% have 3b,3n = A–U for which we failed to obtain a Kt-7 variant structure, so this suggests that it is likely to be another N3-forming 3b,3n sequence. If so that would raise the percentage of N3-forming U4 sequences to 96.4% of the total. In contrast to the other N3-forming k-turns of Kt-46 and the SAM-I riboswitch, the U4 k-turns strongly avoid the 3b = C or 3n = G cells of the array, and the exceptions to this sum to only 1.7% of the total. By contrast 70.7% of the sequences lie on the ascending diagonal, and 62.6% have 3b,3n = G–C which is one of the k-turns least able to undergo metal ion-induced folding. Thus, the U4 snRNA k-turns are N3-forming, but with a strong bias against ion-induced folding.

## DISCUSSION

We have now identified the critical determinant of both folding characteristics and conformation in k-turns. It is the 3b,3n basepair immediately 3′ to the conserved G•A pairs. We had previously shown that this basepair determines whether or not a k-turn will fold in response to the addition of metal ions (11). Perhaps surprisingly, we now show that this single basepair also has a major effect on the structural properties of the k-turn.

Altogether we have analyzed some 30 different sequences, finding an almost perfect correlation between the 3b,3n sequence and the N3/N1 conformation assumed by the k-turn. A given 3b,3n sequence confers the same conformation on different k-turns irrespective of environment including those within the ribosome, in the SAM-I riboswitch and in duplex RNA both free and bound by the L7Ae protein. The only exception is HmKt-7 in the 50S ribosomal subunit, where it is evidently forced into an atypical N1 structure by a combination of tertiary interactions and protein binding. So while the rules correlating 3b,3n sequence and conformation hold true most of the time, a particular environment can overcome the intrinsic preference of a given sequence. However, our data indicate that this is rare, and in general the folding and conformational characteristics follow the properties dictated by the 3b,3n sequence. Moreover, the structures of the HmKt-7 variants inserted into the SAM-I riboswitch demonstrate that changing the 3b,3n sequence is sufficient to switch the equilibrium conformation of the k-turn.

The mechanism by which the 3b,3n sequence exerts its effect on the conformational choice is not fully understood. The 3b,3n pairs either adopt *trans*-Hoogsteen-sugar edge or *cis*-Watson–Crick pairings, yet there is no systematic correlation between this and the resulting k-turn conformation. However, we see there is a good correlation between local helical width at the 2b,2n and 3b,3n pairs. The rotation of the A2b nucleobase from the N3 to the N1 conformation requires the C1'-C1' distance to increase from 8.9 to 11.3 Å on average, and the corresponding distances for the 3b,3n pairs correlate with this. So those associated with the N3 conformation have a mean C1'–C1' distance of 9.6 Å, while for the N1-inducing basepairs this increases to 11.3 Å. This is the most probable origin of the effect, with the 3b,3n pair acting as a transitional element between the 2b,2n G•A pair and the conventional A-form helix of the distal NC helix. In the majority of k-turns, the 4b,4n pair is a standard Watson–Crick basepair, with an average C1'–C1' distance of 10.55 ± 0.18 Å (Supplementary Table S1).

Even without a full molecular explanation of the effects at this time, we can use our data empirically in a predictive way, using the array shown in Figure 4 as a look-up table for k-turn conformation. Perhaps the best evidence that these effects have biological significance comes from the distribution of sequences for particular k-turns across the species. Each k-turn has clearly selected its 3b,3n sequence reflecting specific folding and conformational requirements. Thus, in the *H. marismortui* 50S ribosomal subunit, Kt-46 and Kt-7 both overwhelmingly have selected sequences that fold in response to metal ions, yet the former is N3-forming and the latter N1-forming. At the other extreme, the U4 snRNA k-turn has selected N3-forming 3b,3n sequences, yet in that case it is the subset that also confers an inability to under ion-induced folding. In the mammalian U4/U6.U5 tri-snRNP the k-turn is bound by the 15.5 kDa protein (30), and the properties of this k-turn indicate that it will remain unfolded (and probably a point of flexibility) until this protein binds, perhaps allowing the formation of other interactions. This will likely be important during this stage of the spliceosome cycle.

Considerable efforts currently being made to develop procedures to generate models of unknown RNA structure from sequence, using computer modeling with (31–36) or without (37–42) inclusion of chemical probing data. These methods presently work well in some respects, but perform less well in the prediction of non-standard basepairing. In the most recent *RNA-puzzles* exercise (43) it was noted that the non-Watson–Crick interactions of k-turns were not well handled, and in one case these were modeled using Kt-7 structure. It is highly likely that the distillate of the data represented by Figure 4 can be used predictively on sequences of unknown properties, and could be incorporated into larger-scale modeling procedures. As more and more RNA sequences emerge from genome sequencing and the identification of long non-coding RNA species, this information could provide a valuable guide to probable conformational properties.

## Supplementary Material

Supplementary Data

SUPPLEMENTARY DATA
