# Supplementary material for: A critical base pair in k-turns determines the conformational class adopted, and correlates with biological function
Source: Nucleic Acids Res. 2016 Mar 25;44(11):5390–8. doi: 10.1093/nar/gkw201 (PMC4914095; doi:10.1093/nar/gkw201)
Supplement: Supplementary Data [file gkw201_Supplementary_Data.zip › nar-00403-r-2016-File008.pdf]

A critical base pair in k-turns determines the conformational class adopted, and correlates with biological function

Lin Huang, Jia Wang and David M.J. Lilley

SUPPLEMENTARY INFORMATION

SUPPLEMENTARY FIGURES

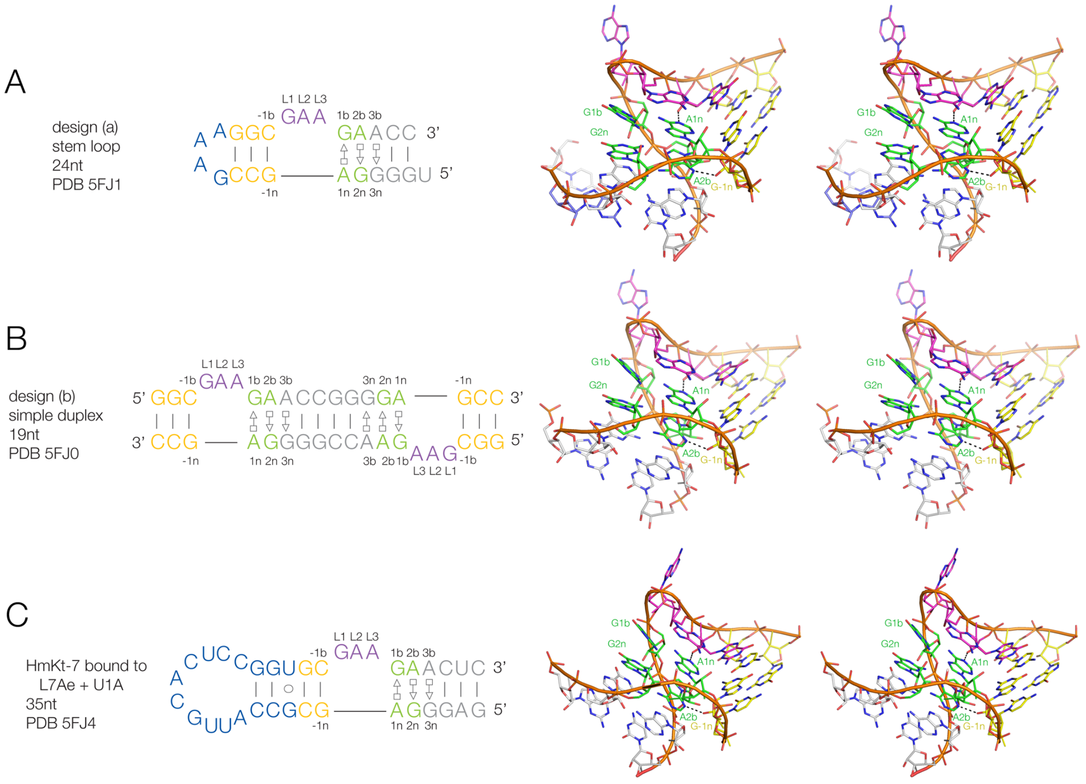

Figure S1. The structures of three forms of HmKt-7. Three different constructs are shown, with the sequence on the left, and the k-turn structure as a parallel-eye stereo pair on the right. **A**. A stem-loop structure, **B**. A simple duplex with two-fold symmetry containing two Kt-7 motifs and **C**. A stem-loop structure bound to L7Ae and U1A proteins. The proteins are not shown in the molecular graphics.

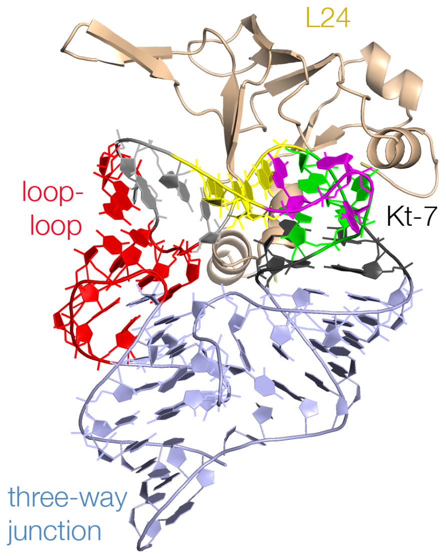

Figure S2. The structural environment of Kt-7 in the *H. marismortui* 50S ribosomal subunit. The local section of 23S rRNA 49-111 is closed by one helix of a three-way helical junction (shown here at the lower end). The longest arm of this junction (on the right-hand side of the image shown) is kinked by Kt-7, the C-helix of which is thus able to make an extensive terminal loop-terminal loop interaction (highlighted red) with the remaining helix (left side). Kt-7 is also bound by the L24 protein. Thus the k-turn is constrained both by tertiary interaction and by protein binding. This parallel-eye stereoscopic image was generated using PDB 3CC2.

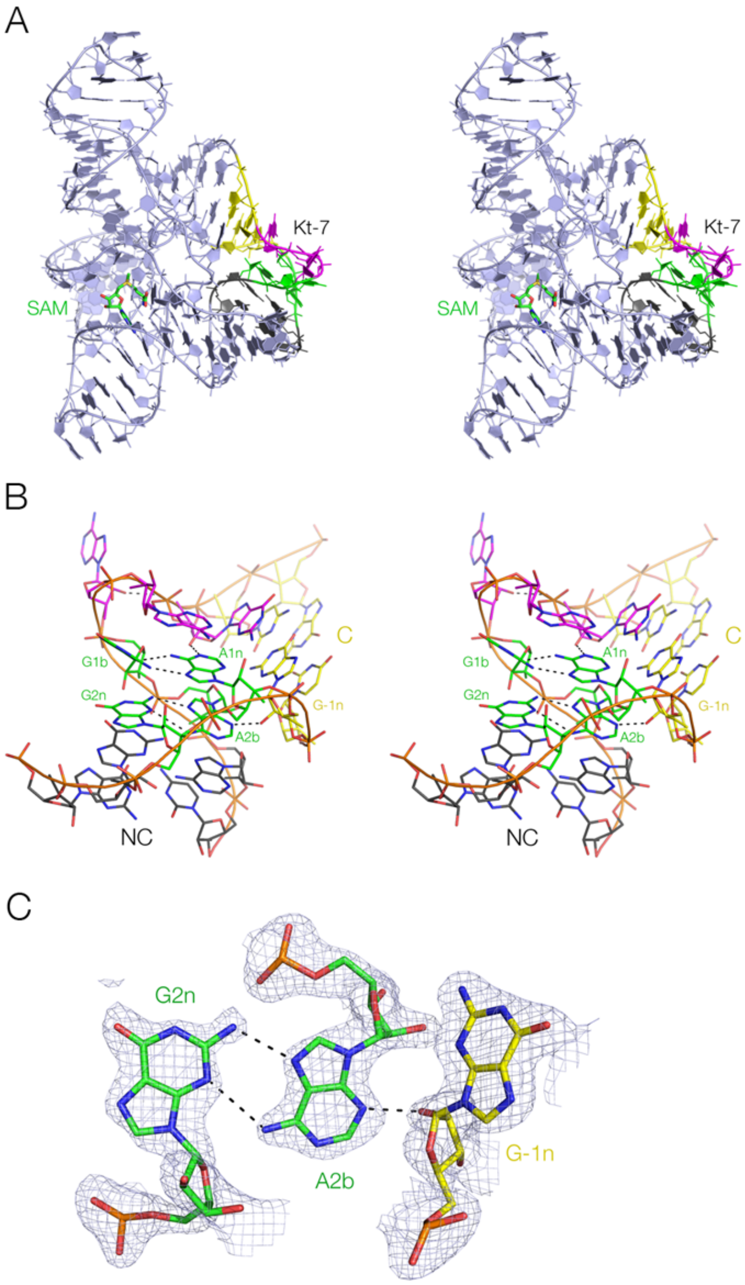

Figure S3. The crystal structure of HmKt-7 (3b,3n = A•G) inserted into the SAM-I riboswitch solved at 1.71 Å resolution (PDB 5FJC). **A**. The structure of the complete riboswitch with the k-turn colored and SAM ligand shown in stick form. **B**. The Kt-7 k-turn structure, with key hydrogen bonds shown. **C**. The G2n•A2b•G-1n triple base interaction with the composite omit 2**F**o-**F**c map shown, contoured at 1 σ. Parts **A** and **B** are shown as parallel-eye stereoscopic pairs.

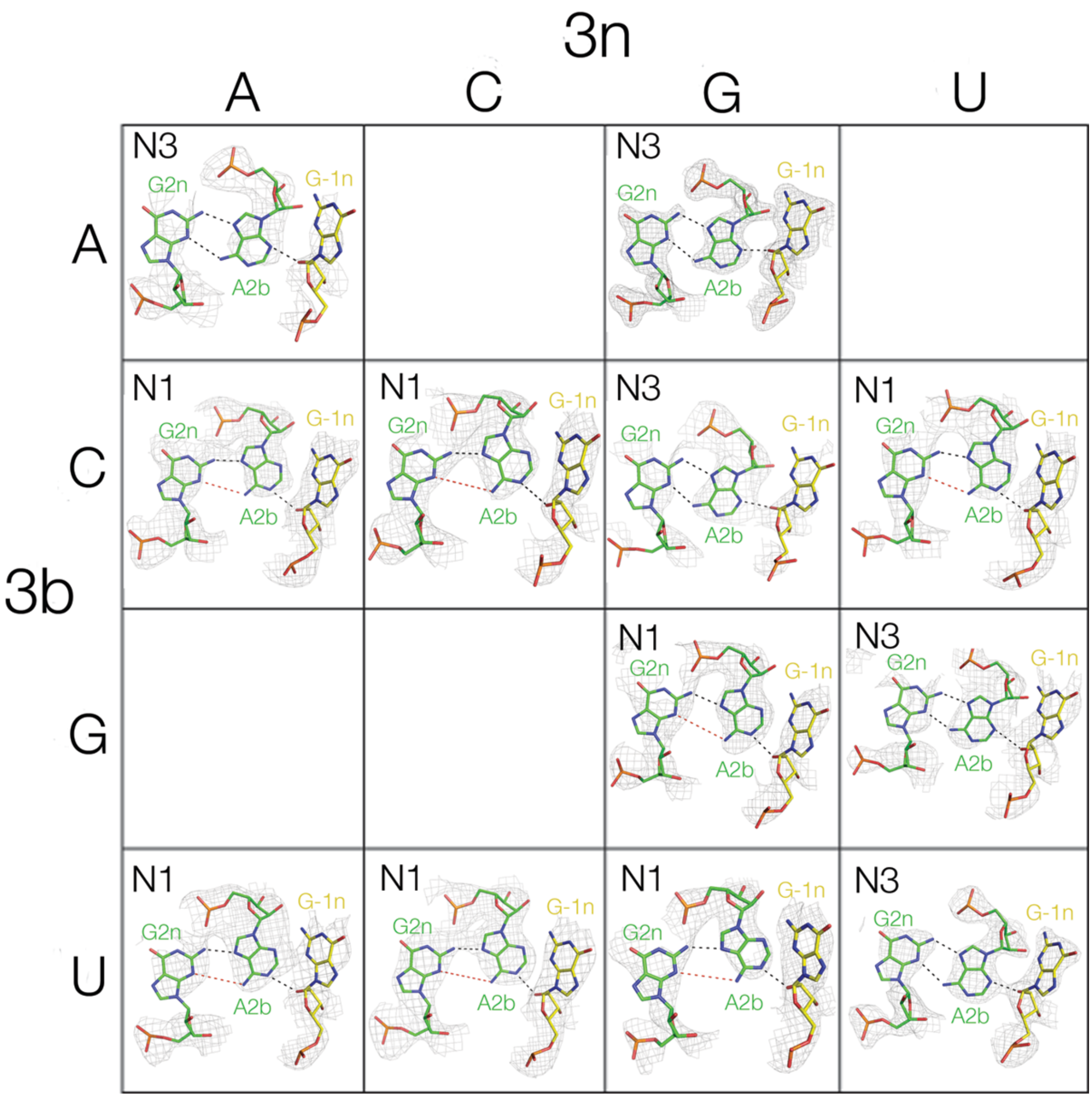

Figure S4. The G2n•A2b•G-1n triple base interactions for the Kt-7 variants in the SAM1 riboswitch context shown in array form as a function of the 3b,3n sequence. The composite omit map is shown for each structure, contoured at 1 σ. G2nN3-A2bN7 distances longer than 3.2 Å (i.e. those in the N1 structures) are shown red, indicating that they are too long to be hydrogen bonded.

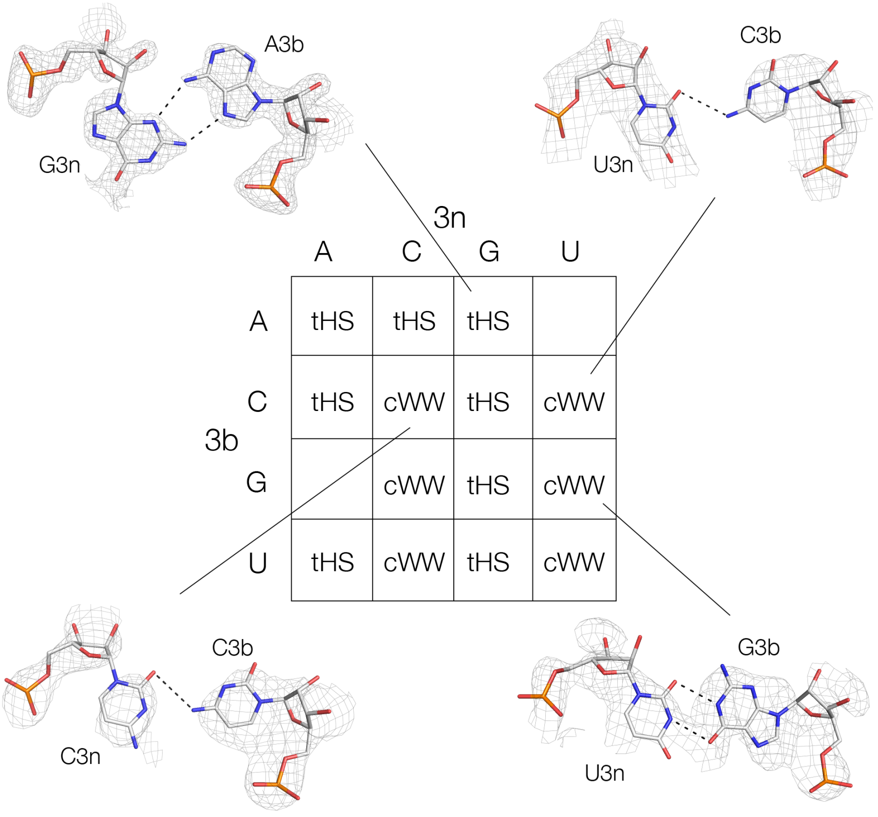

3b \ 3n	A	C	G	U
A	tHS	tHS	tHS	
C	tHS	cWW	tHS	cWW
G		cWW	tHS	cWW
U	tHS	cWW	tHS	cWW

Figure S5. The 3b,3n basepair types for the Kt-7 variants in the SAM1 riboswitch context shown in array form as a function of the 3b,3n sequence. The composite omit map is shown for each structure, are shown for four examples, contoured at 1 σ.

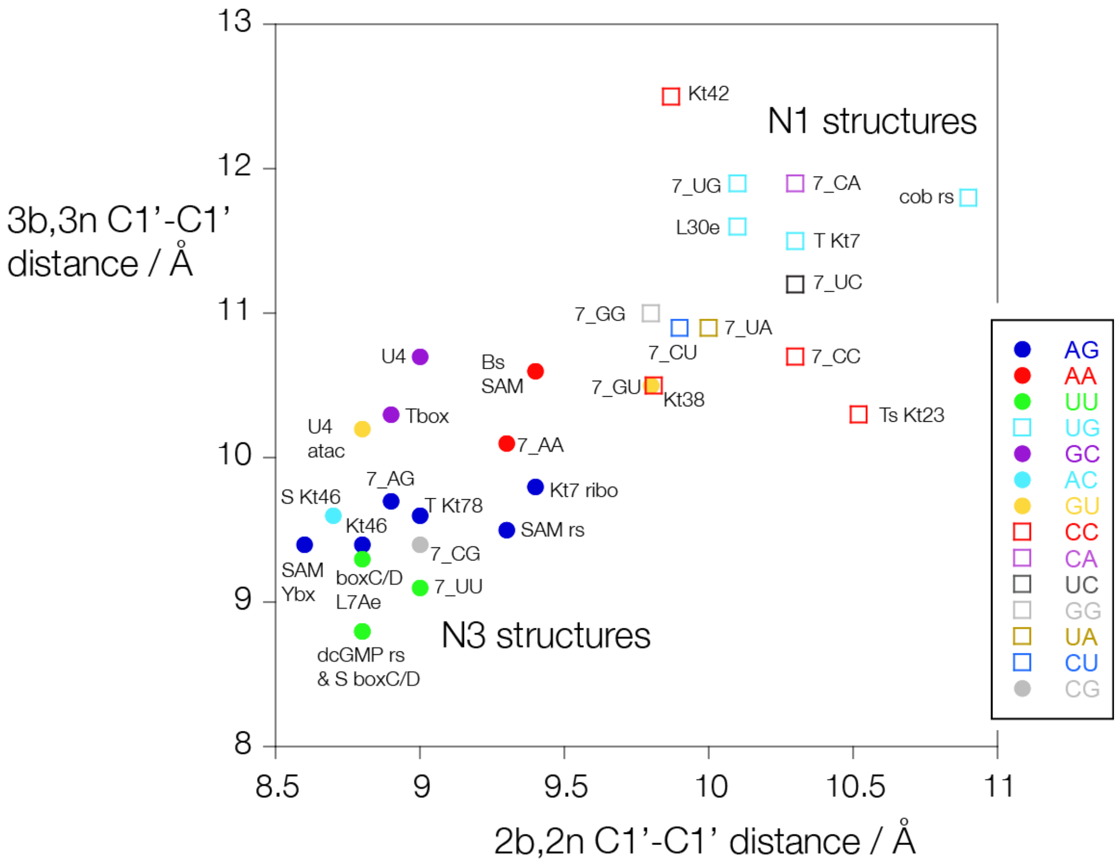

Figure S6. Correlation of C1'-C1' distances for 2b,2n and 3b,3n pairs, plotted to show the 3b,3b basepair in each case. The type of 3b,3b basepair is indicated by the symbol type, according to the key shown on the right, and each point is identified by its k-turn. Abbreviations rs = riboswitch, cob = cobalamine, dcGMP = dicyclic GMP. Ribosomal k-turns have the prefix Kt and modified Kt-7 k-turns are indicated by 7_XX where XX designates the 3b,3n basepair. Kt7 ribo designates the Kt-7 k-turn in the context of the ribosome.

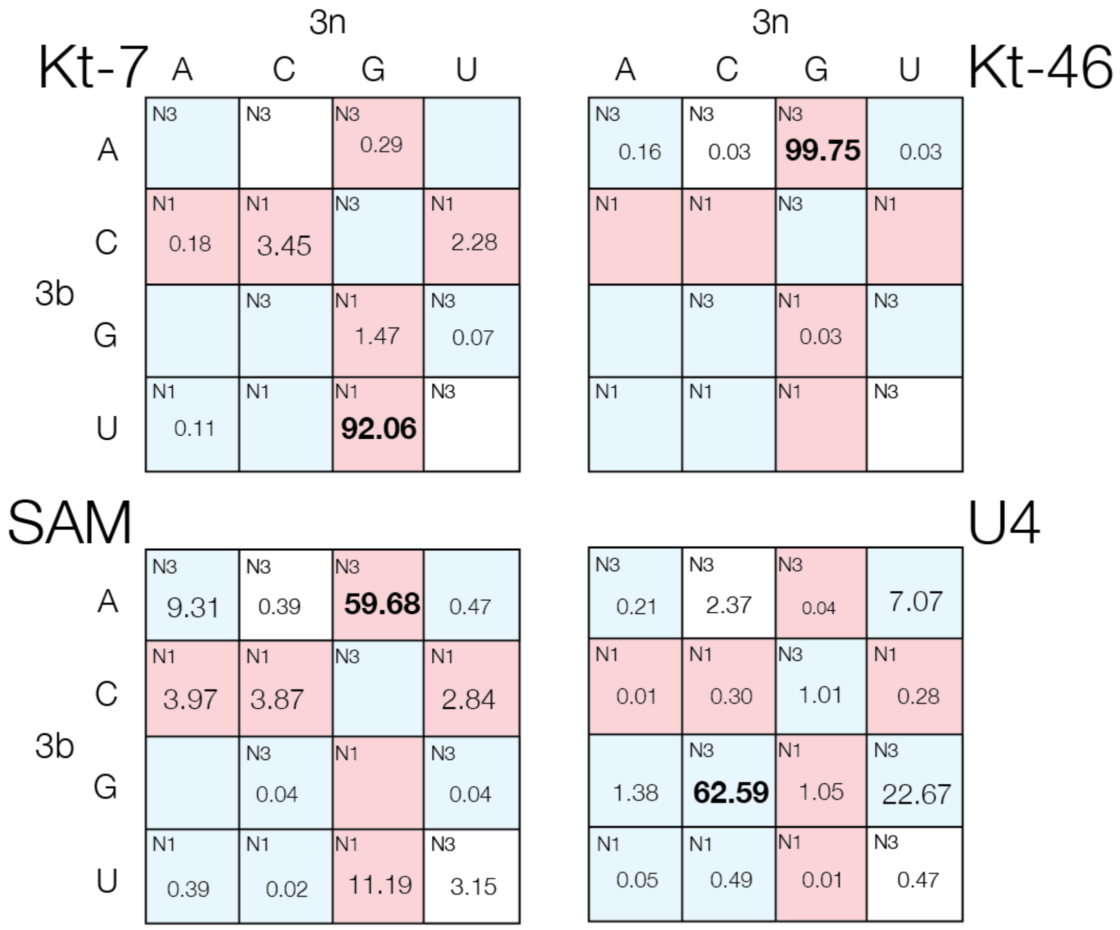

Figure S7. The distribution of natural k-turn sequences for bacterial Kt-7, Kt-46, SAM-I riboswitches and U4 snRNA according to their 3b,3n sequences. Each cell of the arrays is labelled by their preferred N3 or N1 conformation, and colored by their ability to fold in the presence of metal ions (red good folding, blue poor folding). The sequence distribution is shown as percentages of the total for each 3b,3n sequence, with the single largest written bold for each k-turn. The number of sequences analysed were 2,722 bacterial Kt-7, 3,181 Kt-46, 4,755 SAM-I riboswitch and 9,235 U4 snRNA k-turns.

SUPPLEMENTARY TABLES

**Name**	**PDB**	**C**	**L1-L3**	**NC**	**res**	**space group**	**3bn**	**conf**	**1b1n**	**2b2n**	**3b3n**	**4b4n**	**3b3n conf**
									**C1'-C1' / Å**				
**SAMI**	3GX5	CC	GAC	GAAA	2.40	$P4_32_12$	AG	N3	8.9	9.3	9.5	10.6	tHS
**rsw**		GG		AGGU									
**SAMI rsw**	3V7E	CC	GAU	GAAA	2.80	C2	AG	N3	9.3	8.6	9.4	10.6	tHS
**YbxF**		GG		AGGU									
**SAMI rsw**	4KQY	CC	GAC	GAAG	3.02	$P3_121$	AA	N3	10.1	9.4	10.6	10.9	tHS
***B. subtilis* yitJ**		GG		AGAC									
**c-di-GMP-II**	3Q3Z	AC	AAU	GAUG	2.51	$P2_1$	UU	N3	9.1	8.8	8.8	10.4	cWW
**rsw**		UG		AGUU									
**cobalamine**	4GXY	GC	GAG	GAUC	3.05	$P3_121$	UG	N1	9.0	10.9	11.8	10.4	tHS
**rsw**		UG		AGGG									
**T-box rsw**	4LCK	GC	GAU	GAGA	3.20	$C222_1$	GC	N3	9.4	8.9	10.3	10.8	cWW
**YbxF**		CG		AGCU									
**HmKt-7**	3CC2	GC	GAA	GAAC	2.40	$C222_1$	AG	N1	9.2	9.4	9.8	10.4	tHS
		CG		AGGG									
**TtKt-7**	4Y4O	GC	GUG	GAUC	2.30	$P2_12_12_1$	UG	N1	9.2	10.3	11.5	10.5	tHS
		CG		AGGG									
**DrKt-7**	2ZJR	GC	UUU	GACU	2.91	I222	CC	N1	9.1	10.2	9.6	10.5	cWW
		CG		AGCA									
**HmKt-46**	3CC2	GC	GAA	GAAC	2.40	$C222_1$	AG	N3	9.1	8.8	9.4	10.3	tHS
		CG		AGGG									
**ScKt-46**	3U5D	GU	GAC	GAAG	3.00	$P2_1$	AC	N3	9.1	8.7	9.6	10.3	tHS
	4U4R	CA		AGCU	2.80				9.1	8.6	9.6	10.3	tHS
**TtKt-78**	3UMY	CC	UGU	GAAC	1.90	$P2_12_12_1$	AG	N3	8.7	9.0	9.6	10.5	tHS
		GG		AGGG									
**box C/D**	1RLG	GG	CGU	GAUG	2.70	P23	UU	N3	9.4	8.8	9.3	10.6	cWW
**AfL7Ae**		CC		AGUC									
**Box C/D**	3PLA	CU	UGU	GAUG	3.15	$P4_12_12$	UU	N3	9.2	8.8	8.8	10.6	cWW
**SsL7Ae**		GA		AGUC									
**pre-mRNA**	1T0K	GC	AGA	GAUG	3.24	$P4_12_12$	UG	N1	9.6	10.1	11.6	10.5	tHS
**ScL30e**		UG		AGGC									
**HsU4**	1E7K	CC	AAU	GAGG	2.90	$P2_12_12_1$	GC	N3	9.6	9.0	10.7	10.9	cWW
**15.5KD**		GG		AGCC									
**HsU4**	2OZB	CC	AAU	GAGG	2.60	$P2_12_12_1$	GC	N3	9.1	8.7	10.6	10.7	cWW
**Prp31+15.5KD**		GG		AGCC									
**HsU4atac**	3SIU	CC	AAU	GAGC	2.63	$P2_1$	GU	N3	8.9	8.8	10.2	10.6	cWW
**Prp31+15.5KD**		GG		AGUC									

Table S1. Structural properties of the known natural k-turns. For each is shown the PDB identifier, the sequence of the C helix (*C*), loop (*L1-L3*) and NC helix (*NC*), the resolution of the crystal structure (*res*, given in Å), the crystal space group (*space group*), the 3b,3n sequence (*3bn*) the N3 or N1 conformation (*conf*), the C1'-C1' distances for the 1b,1n, 2b,2n, 3b,3n and 4b,4n basepairs (each given in Å) and finally the type of basepair for 3b,3n (*3b3n conf*) as either *trans*-Hoogsteen-sugar (*tHS*) or *cis*-Watson-Crick (*cWW*) pairs.

environment :	simple duplex	simple duplex *	stem loop *	bound to L7Ae	bound to L7Ae + U1A*	SAMI rsw	SAMI rsw *	ribosome
PDB identifier	4C40 4CS1	5FJ0	5FJ1	4BW0	5FJ4	4B5R	5FJC	3CC2
space group	$P6_322$	$P4_222$	$P2_12_12_1$	$P4_32_12$	$C222_1$	$P4_32_12$	$P4_32_12$	$C222_1$
resolution /Å	2.2, 2.0	2.2	2.9	2.33	3.10	2.95	1.71	2.4
RNA in ASU	1	3	8	1	2	1	1	1
conformation	N3	N3	N3	N3	N3	N3	N3	N1

Table S2. Summary of independent crystal structures of Kt-7 determined in different structural environments. Structures determined in this study are indicated by an asterisk.

RMSD	4CS1	5FJ0A	5FJ0B	5FJ0C	5FJ1A	5FJ1B	5FJ1C	5FJ1D	5FJ1E	5FJ1F	5FJ1G	5FJ1H	4BW0	5FJ4A	5FJ4B	4B5R	5FJC	3CC2
**4CS1**	0	0.488	0.363	0.410	0.306	0.299	0.310	0.301	0.303	0.313	0.302	0.305	0.639	0.544	0.623	0.389	0.409	1.384
**5FJ0A**	0.488	0	0.194	0.258	0.288	0.264	0.267	0.262	0.263	0.267	0.264	0.253	0.445	0.289	0.317	0.534	0.564	1.492
**5FJ0B**	0.363	0.194	0	0.140	0.314	0.346	0.342	0.364	0.368	0.375	0.343	0.346	0.657	0.567	0.649	0.379	0.470	1.526
**5FJ0C**	0.410	0.258	0.140	0	0.177	0.161	0.172	0.186	0.171	0.173	0.185	0.188	0.545	0.432	0.493	0.376	0.423	1.596
**5FJ1A**	0.306	0.288	0.314	0.177	0	0.077	0.116	0.154	0.129	0.120	0.134	0.105	0.680	0.471	0.557	0.350	0.444	1.533
**5FJ1B**	0.299	0.264	0.346	0.161	0.077	0	0.095	0.100	0.103	0.100	0.108	0.077	0.649	0.446	0.533	0.364	0.468	1.590
**5FJ1C**	0.310	0.267	0.342	0.172	0.116	0.095	0	0.091	0.050	0.083	0.087	0.093	0.604	0.385	0.484	0.381	0.515	1.665
**5FJ1D**	0.301	0.262	0.364	0.186	0.154	0.100	0.091	0	0.088	0.070	0.059	0.083	0.603	0.404	0.481	0.410	0.534	1.650
**5FJ1E**	0.303	0.263	0.368	0.171	0.129	0.103	0.050	0.088	0	0.072	0.088	0.088	0.608	0.391	0.483	0.392	0.520	1.658
**5FJ1F**	0.313	0.267	0.375	0.173	0.120	0.100	0.083	0.070	0.072	0	0.074	0.065	0.632	0.399	0.496	0.399	0.518	1.657
**5FJ1G**	0.302	0.264	0.343	0.185	0.134	0.108	0.087	0.059	0.088	0.074	0	0.081	0.606	0.407	0.491	0.404	0.522	1.651
**5FJ1H**	0.305	0.253	0.346	0.188	0.105	0.077	0.093	0.083	0.088	0.065	0.081	0	0.616	0.407	0.498	0.391	0.514	1.666
**4BW0**	0.639	0.445	0.657	0.545	0.680	0.649	0.604	0.603	0.608	0.632	0.606	0.616	0	0.501	0.465	0.868	0.987	1.853
**5FJ4A**	0.544	0.289	0.567	0.432	0.471	0.446	0.385	0.404	0.391	0.399	0.407	0.407	0.501	0	0.347	0.585	0.739	1.818
**5FJ4B**	0.623	0.317	0.649	0.493	0.557	0.533	0.484	0.481	0.483	0.496	0.491	0.498	0.465	0.347	0	0.699	0.848	1.812
**4B5R**	0.389	0.534	0.379	0.376	0.350	0.364	0.381	0.410	0.392	0.399	0.404	0.391	0.868	0.585	0.699	0	0.387	1.433
**5FJC**	0.409	0.564	0.470	0.423	0.444	0.468	0.515	0.534	0.520	0.518	0.522	0.514	0.987	0.739	0.848	0.387	0	1.180
**3CC2**	1.384	1.492	1.526	1.596	1.533	1.590	1.665	1.650	1.658	1.657	1.651	1.666	1.853	1.818	1.812	1.433	1.180	0

Table S3. Comparison of Kt-7 structures in different crystal environments. RMSD values (Å) are shown for each pairwise comparison of structures, indicated by their PDB identifiers.

PDB	res	space	3bn	conf	1b1n	2b2n	3b3n	4b4n	3b3n
		**group**			**C1'-C1'**				**base pair**
**5FJC**	1.71	$P4_32_12$	**AG**	N3	8.8	8.9	9.7	10.6	tHS
**5FK2**	2.60	$P4_32_12$	**GG**	N1	9.6	9.8	11.0	10.7	tHS
**5FK1**	2.50	$P4_32_12$	**UG**	N1	9.8	10.1	11.9	10.6	tHS
**5FK3**	2.50	$P4_32_12$	**CC**	N1	9.4	10.3	10.7	10.6	cWW
**5FK4**	2.43	$P4_32_12$	**UU**	N3	8.8	9.0	9.1	10.4	cWW
**5FK5**	3.31	$P4_32_12$	**AA**	N3	9.1	9.3	10.1	10.7	tHS
**5FK6**	2.50	$P4_32_12$	**CA**	N1	9.3	10.3	11.9	10.9	tHS
**5FKD**	3.00	$P4_32_12$	**UA**	N1	9.5	10.0	10.9	10.7	tHS
**5FKF**	2.80	$P4_32_12$	**UC**	N1	9.4	10.3	11.2	10.7	cWW
**5FKG**	2.95	$P4_32_12$	**CG**	N3	8.9	9.0	9.4	10.3	tHS
**5FKH**	2.65	$P4_32_12$	**CU**	N1	9.7	9.9	10.9	10.7	cWW
**5FKE**	2.80	$P4_32_12$	**GU**	N3	9.4	9.8	10.5	10.8	cWW

Table S4. Structural properties of Kt-7 variants within the SAM-I riboswitch as a function of 3b,3n sequence. For each is shown the resolution of the crystal structure (*res*, given in Å), the crystal space group (*space group*), the 3b,3n sequence (*3bn*) the N3 or N1 conformation (*conf*), the C1'-C1' distances for the 1b,1n, 2b,2n, 3b,3n and 4b,4n basepairs (each given in Å) and finally the type of basepair for 3b,3n (*3b3n conf*) as either *trans*-Hoogsteen-sugar (*tHS*) or *cis*-Watson-Crick (*cWW*) pairs.

	Simple duplex	Stem loop	Bound to L7Ae and U1A
PDB identifier	5FJ0	5FJ1	5FJ4
	65218	65223	65224
**Data collection**			
Space group	$P4_222$	$P2_12_12_1$	$C222_1$
Cell dimensions			
a, *b*, *c* (Å)	50.50, 50.50, 144.95	84.05, 84.36, 174.50	131.85, 161.23, 149.90
α, β, γ (°)	90, 90, 90	90, 90, 90	90, 90, 90
Resolution (Å)	48.32 - 2.20 (2.28 - 2.2)	38.72 - 2.75 (2.90 - 2.75)	84.37 - 2.95 (3.09 - 2.95)
R_{merge}	0.038 (1.890)	0.056 (0.686)	0.081 (1.377)
I / σI	26.6 (1.3)	12.4 (1.9)	23.6 (1.1)
CC(1/2)	0.999 (0.520)	0.997 (0.533)	0.998 (0.404)
Completeness (%)	100.00 (100.00)	99.2 (98.5)	99.9 (99.7)
Redundancy	10.2 (10.2)	4.4 (4.3)	7.5 (7.6)
**Refinement**			
Resolution (Å)	47.69 - 2.20 (2.52 - 2.20)	38.72 - 2.75 (2.83 - 2.75)	80.61 - 2.95 (3.04 - 2.95)
No. reflections	10187	32687	33933
R_{work} / R_{free}	0.205 / 0.237	0.168 / 0.183	0.193 / (0.240)
No. atoms			
Macromolecules	1242	4184	6475
Ligand/ion	3	24	-
Water	-	16	-
B-factors			
Macromolecules	92.7	77.52	128.18
Ligand/ion	95.7	91.80	-
Water	-	67.36	-
R.m.s deviations			
Bond lengths (Å)	0.002	0.002	0.016
Bond angles	0.42	0.58	1.96
Ramachandran			
Favored and allowed			97.6
outliers			2.6

Table S5. Crystallographic data collection and refinement statistics for HmKt-7 determined in different environments.

3b,3n	AG	GG	UG	CC	UU	AA	CA	UA
PDB identifier	5FJC	5FK2	5FK1	5FK3	5FK4	5FK5	5FK6	5FKD
Data collection	65237	65249	65250	65293	65294	65295	65296	65297
Space group	$P4_32_12$	$P4_32_12$	$P4_32_12$	$P4_32_12$	$P4_32_12$	$P4_32_12$	$P4_32_12$	$P4_32_12$
Cell dimensions								
a, *b*, *c* (Å)	60.40, 60.40, 152.95	61.48, 61.48, 153.75	62.58, 62.58, 154.73	62.26, 62.26, 153.78	57.55, 57.55, 155.62	61.78, 61.78, 154.98	62.17, 62.17, 153.36	61.77, 61.77, 153.93
α, β, γ (°)	90, 90, 90	90, 90, 90	90, 90, 90	90, 90, 90	90, 90, 90	90, 90, 90	90, 90, 90	90, 90, 90
Resolution (Å)	41.13 - 1.71 (1.77 - 1.71)	76.87 - 2.60 (2.72 - 2.60)	77.36- 2.50 (2.60 - 2.50)	76.89 - 2.50 (2.60 - 2.50)	39.37 - 2.43 (2.49 - 2.43)	28.99 - 3.31 (3.40 - 3.31)	62.17 - 2.50 (2.60 - 2.50)	61.77 - 2.80 (2.95 - 2.80)
R_{merge}	0.048 (1.598)	0.068 (0.976)	0.060 (1.611)	0.092 (1.306)	0.064 (0.639)	0.143 (1.367)	0.065 (2.390)	0.194 (2.675)
I / σI	20.6 (1.2)	13.2 (2.1)	11.3 (1.1)	13.1 (1.9)	19.2 (2.9)	11.9 (2.7)	13.1 (1.0)	8.2 (1.2)
CC(1/2)	0.999 (0.549)	0.998 (0.783)	0.996 (0.604)	0.932 (0.634)			0.995 (0.707)	0.998 (0.302)
Completeness (%)	99.7 (97.0)	100 (100)	99.8 (99.7)	100 (100)	99.9 (100)	99.8 (99.1)	99.7 (100)	100 (100)
Redundancy	9.6 (7.4)	8.3 (8.3)	5.6 (5.8)	11.3 (11.3)	7.5 (8.0)	9.2 (9.5)	8.8 (9.2)	14.5 (14.2)
**Refinement**								
Resolution (Å)	41.13 - 1.71 (1.74 - 1.71)	48.01 - 2.60 (2.69 - 2.60)	58.01 - 2.50 (2.59 - 2.50)	57.71 - 2.50 (2.62 - 2.50)	39.37 - 2.43 (2.58 - 2.43)	28.99 - 3.31 (3.52 - 3.31)	57.62 - 2.50 (2.63 - 2.50)	57.34 - 3.00 (3.30 - 3.00)
No. reflections	58114 31391	17286 9656	20018 11185	19939 11084	18742 10495	8464 4859	19660 10933	11344 6437
R_{work} / R_{free}	0.203 / 0.241	0.228 / 0.269	0.241 / 0.289	0.210 / 0.268	0.193 / 0.248	0.204 / 0.249	0.217 / 0.276	29.3 / 23.6
No. atoms								
Macromolecules	2102	2061	2057	2077	2031	2059	2055	2057
Ligand/ion	56	11	15	18	17	14	15	14
Water	183	14	15	12	34	-	3	-
B-factors								
Macromolecules	40.85	106.28	116.41	87.25	58.68	111.75	134.07	120.63
Ligand/ion	58.42	163.27	162.93	153.71	88.49	-	203.86	227.49
Solvent	42.59	61.82	62.47	57.56	31.66	-	60.73	-
R.m.s deviations								
Bond lengths (Å)	0.005	0.002	0.002	0.002	0.008	0.003	0.002	0.002
Bond angles (°)	1.14	0.65	0.64	0.68	1.46	0.718	0.62	0.61

	UC	CG	CU	GU
PDB identifier	5FKF	5FKG	5FKH	5FKE
**Data collection**	65298	65299	65300	65301
Space group	$P4_32_12$	$P4_32_12$	$P4_32_12$	$P4_32_12$
Cell dimensions				
a, *b*, *c* (Å)	61.81, 61.81,	61.25, 61.25,	62.50, 62.50,	62.67, 62.67,

	154.94	155.97	155.10	154.43
α, β, γ (°)	90, 90, 90	90, 90, 90	90, 90, 90	90, 90, 90
Resolution (Å)	77.47 - 2.80 (2.95 - 2.80)	61.25 - 2.95 (3.13 - 2.95)	42.50 - 2.65 (2.65 - 2.78)	77.21 - 2.60 (2.72 – 2.60)
R_{merge}	0.089 (0.525)	0.157 (1.551)	0.031 (0.553)	0.109 (2.584)
I / σI	10.5 (2.9)	9.7 (1.9)	16.6 (1.7)	8.3 (0.8)
CC(1/2)	0.994 (0.930)	0.969 (0.644)	0.987 (0.842)	0.989 (0.549)
Completeness (%)	100 (100)	100 (100)	98.7 (99.7)	99.9 (100)
Redundancy	6.2 (6.3)	9.5 (10 .1)	3.1 (3.3)	9.9 (10.6)
**Refinement**				
Resolution (Å)	57.42 - 2.80 (3.02 - 2.80)	57.01 - 2.95 (3.18 - 2.95)	42.50 - 2.65 (2.85 – 2.65)	58.08 - 2.80 (3.02 - 2.80)
No. reflections	14070 7923	11920 6751	15713 9323	14266 8015
R_{work} / R_{free}	0.207 / 0.257	0.237 / 0.299	0.233 / 0.278	0.250 / 0.306
No. atoms				
Macromolecules	2058	2058	2055	2058
Ligand/ion	19	19	15	19
Water		-	5	-
B-factors				
Macromolecules	76.65	114.48	133.86	175.25
Ligand/ion	138.70	161.61	181.82	285.93
Solvent		-	61.46	-
R.m.s deviations				
Bond lengths (Å)	0.005	0.005	0.002	0.003
Bond angles (°)	0.99	1.21	0.62	0.94

Table S6 Crystallographic data collection and refinement statistics for HmKt-7 variants in SAM-I riboswitch.

MATERIALS AND METHODS

RNA synthesis

Ribooligonucleotides were synthesized using *t*-BDMS phosphoramidite chemistry (Beaucage & Caruthers, 1981), as described in Wilson et al. (Wilson et al, 2001). Oligoribonucleotides were deprotected in 25% ethanol/ammonia solution at 25°C for 2 h, and evaporated to dryness. Oligoribonucleotides were redissolved in 100 μl DMSO to which was added 125 μL 1 M triethylamine trihydrofluoride (TEA, 3HF) (Sigma-Aldrich) and incubated at 65°C for 2.5 h to remove *t*-BDMS protecting groups. All oligonucleotides were purified by gel electrophoresis in polyacrylamide in the presence of 7 M urea, and the full length RNA product was visualized by UV shadowing. The band was excised and electroeluted using an Elutrap (Whatman) into 45 mM Tris.borate (pH 8.5), 5 mM EDTA buffer for 8 h at 200 V at 4°C. The RNA was precipitated with ethanol, washed once with 70 % ethanol and dissolved in water. The concentration of RNA was determined by measuring the absorbance at 260 nm.

Preparation of SAM-I riboswitch variants

A plasmid containing a gene encoding the *Thermoanaerobacter tengcongensis* SAM-I riboswitch (Montange & Batey, 2006) in which the natural k-turn was replaced by HmKt-7 (Daldrop & Lilley, 2013) was supplied by Dr. P. Daldrop. Substitutions at the 3b,3n position were generated by site-directed mutagenesis. The SAM-I riboswitch variants were transcribed from PCR-amplified templates, and purified by gel electrophoresis under denaturing conditions. The PCR reaction contained 20 mM Tris-HCl (pH 8.8), 10 mM $(NH_4)_2SO_4$, 10 mM KCl, 2 mM $MgSO_4$, 0.1 % Triton X-100, 0.2 mM of each dNTP, 1 μM each primer, 1 U/mL Taq polymerase (New England Biolabs), 600 ng/ml SAM-I plasmid. Transcription reactions contained 40 mM Tris- HCl (pH 8.0), 2 mM of each NTP (Sigma), 30 mM $MgCl_2$, 10 mM DTT, 3.2 mM spermidine, 0.01% Triton X-100, 0.25 mg/ml T7-RNA polymerase, 10.0% (v/v) DNA template from the PCR reaction and 0.1 U/mL pyrophosphatase (Sigma). Reactions were incubated at 37°C for 4 h and precipitated with 3 reaction volumes of ethanol. Transcribed RNA was purified by electrophoresis in 10% polyacrylamide gels (19:1), in 90 mM Tris.borate (pH 8.3), 10 mM EDTA, 7 M urea. The full length RNA product was visualized by UV shadowing, bands were excised and electroeluted using an Elutrap (Whatman) into 45 mM Tris.borate (pH 8.5), 5 mM EDTA buffer for

8 h at 200 V at 4°C. The RNA was precipitated with ethanol, washed once with 70 % ethanol and dissolved in water. The concentration of RNA was determined by measuring the absorbance at 260 nm using extinction coefficients calculated from the nucleotide composition and a correction factor for the hypochromic effect.

Expression and purification of human U1 snRNP protein A

U1A-RBD (residues 1-102) (Nagai et al, 1990) was expressed in the *Escherichia coli* BL21-Gold (DE3) pLysS cells (Stratagene) using a T7 RNA polymerase expression vector. Freshly-transformed colonies were picked and grown in 6 L LB medium and induced with 0.2 mM IPTG at 37 °C for 4 h. Harvested cells were resuspended in 20 mM Tris.HCl (pH 8.0), 50 mM NaCl, 1 mM phenylmethylsulfonyl fluoride (PMSF) (buffer T) and lysed by sonication. Cell debris was removed by centrifugation. U1A was applied to three tandem 5 ml CM columns (GE Healthcare) and the protein eluted with 200 mM NaCl in buffer T. U1A was then applied to a heparin column (GE Healthcare) and eluted at 400 mM NaCl using a gradient from 50 to 2000 mM NaCl in 20 mM HEPES.Na (pH 7.6). The protein was further purified using a Superset 75 gel filtration column in a buffer containing 5 mM Tris.HCl (pH 8.0), 100 mM NaCl.

Expression and purification of *A. fulgidus* L7Ae

The gene encoding full-length *A. fulgidus* L7Ae was cloned into a modified pET-Duet1 plasmid (Novagen) (Huang et al, 2011) using the *Hin*dIII and *Eco*RI sites. The L7Ae gene was fused upstream of a hexahistidine-encoding sequence with a PreScission-cleavable linker. The hexahistidine-L7Ae fusion protein was expressed in *E. coli* BL21-Gold (DE3) pLysS cells (Stratagene) induced with 0.2 mM IPTG at 20°C for 12 h. Harvested cells were resuspended in 20 mM Tris-HCl (pH 8.0), 500 mM NaCl, 10 mM imidazole, 1 mM PMSF (buffer A) and lysed by sonication. The protein suspension was heated at 85°C for 20 min. in the presence of 10 mM $MgCl_2$ to denature endogenous protein and this was removed by centrifugation at 18,000 rpm for 30 min at 4°C. L7Ae was applied to a HisTrap column (GE Healthcare), washed with 25 mM imidazole in buffer A, and the protein was eluted with 500 mM imidazole in buffer A. The six-His tag was cleaved from L7Ae by incubation with PreScission protease in 20 mM HEPES-Na (pH 7.6), 100 mM NaCl, 0.5 mM EDTA at 4-8°C for 16 h. L7Ae was applied to a heparin column (GE Healthcare) and eluted at 250 mM NaCl in a gradient from 50 to 2,000 mM NaCl in 20 mM HEPES-Na (pH

7.6). The protein was further purified using a Superdex 200 gel filtration column in a buffer containing 5 mM Tris.HCl (pH 8.0), 100 mM NaCl. The protein concentration was measured by absorbance at 280 nm using a molar extinction coefficient of 5,240 M^{-1} cm^{-1} for L7Ae. The protein was concentrated to 20 mg/ml in buffer containing 5 mM Tris-HCl (pH 8.0), 100 mM NaCl, and stored at - 20 °C as aliquots.

Crystallization, structure determination, and refinement

Kt-7 determined in different structural environments

Two types of design were used in crystallization trials of Kt-7 RNA in the absence of protein. These were **(a)** a 24 nt RNA with a GAAA tetraloop closing the C-helix, and **(b)** a 19 nt RNA oligonucleotide with two inverted copies of the Kt-7. A solution of 1 mM RNA in 5 mM Tris.HCl (pH 8.0), 100 mM NaCl was heated to 95 °C for 1 min, and slowly cooled to 20°C. $MgCl_2$ was then added to a final concentration of 10 mM. Crystallization was performed using the hanging-drop vapor diffusion method.

Stem loop (a) - corresponding to PDB 5FJ1. An aliquot of 1.0 µL of prefolded RNA was mixed with an equal volume of well solution comprising 60% v/v Tacsimate buffer (pH 7.0) at 20 °C. Crystals of space group $P2_12_12_1$ appeared after 10 days. The crystals were directly flash frozen by mounting in nylon loops and plunging into liquid nitrogen. A 2.75 Å resolution dataset was collected on beamline I03 of the Diamond Light Source. The crystals had unit cell dimensions *a* = 84.1 Å, *b* = 84.4 Å, *c* = 174.5 Å. The molecular weight of a molecule is 7.9 kDa, and the asymmetric unit contained eight independent molecules. The structure was determined by molecular replacement using the program PHASER (McCoy et al, 2007) with *H. marismortui* Kt-7 (PDB 4BW0) as the search model. The remaining nucleotides were added to the model based on inspection of electron and difference density maps.

Simple duplex (b) - corresponding to PDB 5FJ0. An aliquot of 1.0 µL of prefolded RNA was mixed with an equal volume of well solution comprising 40 mM $MgCl_2$, 0.05 M sodium cacodylate (pH 6.0) and 5% v/v 2-methan-2,4-pentanediol (MPD) at 20 °C. Crystals of space group $P4_222$ appeared after 20 days. Crystals were briefly washed in well solution supplemented with 30% v/v MPD. The crystals were flash frozen by mounting in nylon loops and plunging into liquid nitrogen. A 2.2 Å resolution dataset was collected on beamline ID29 at the European Synchrotron Radiation Facility (ESRF). The crystals had unit cell dimensions *a* = 50.5 Å, *b* = 50.5

Å, *c* = 145.0 Å. The molecular weight of a molecule is 6.3 kDa, and the asymmetric unit contained three molecules. The structure was determined by molecular replacement using the program PHASER (McCoy et al, 2007) with *H. marismortui* Kt-7 (PDB 4CS1) as the search model.

Kt-7 bound to L7Ae and U1A - corresponding to PDB 5FJ4. A mixture of 0.25 mM RNA, 0.25 mM U1A-RBD and 0.25 mM L7Ae in 5 mM Tris.HCl (pH 8.0), 100 mM NaCl, 10 mM $MgCl_2$ was incubated for 5 min at 37°C. Crystals were grown by vapor diffusion using drops prepared by mixing 1.0 µL of the RNA–protein complex with 1 µL of a reservoir solution comprising 100 mM Tris.HCl (pH 8.5), 2.0 M ammonium dihydrogen phosphate at 7°C. Crystals (100-300 µm) appeared after 5 days. They were transferred to a solution containing 100 mM Tris.HCl (pH 8.4), 4 M sodium formate and 5% glycerol for ~ 10 s. The crystals were flash frozen by mounting in nylon loops and plunging into liquid nitrogen. The crystals were characterized in-house using a MicroMax HF007 copper rotating-anode X-ray generator equipped with an ACTOR sample changer system and a Saturn 944HG+ CCD detector (Rigaku). Suitable samples were stored and subsequently used to measure full datasets on beamline I03 of the Diamond Light Source. The crystals had space group $C222_1$ and unit cell dimensions *a* = 131.9 Å, *b* = 161.2 Å, *c* = 149.9 Å. The structure was determined by molecular replacement using the program PHASER (McCoy et al, 2007) with PDB 4C4W as the search model. Ramachandran analysis shows that 97.6% of amino acid residues are in the most favored and additionally allowed regions. Model geometry and the fit to electron-density maps were monitored with MOLPROBITY (Chen et al, 2010) and the validation tools in COOT. Crystallographic statistics for HmKt-7 are presented in Table S5.

The SAM-I riboswitch variants

The SAM-I riboswitch variants (Table S4) were crystallized using the hanging drop method. Crystal trays were set up by mixing 1 µL of mother liquor with 1 µL of 400 µM RNA plus 1 mM S-adenosylmethionine (Sigma Aldrich) in 40 mM Na-cacodylate (pH 7.0). Drops were seeded using a micro-crystals taken from crystal trays containing the unmodified RNA (corresponding to structure PDB 4B5R). The mother liquor of the drop that yielded the crystal variants used for data collection contained 40 mM Na-cacodylate (pH 7.0), 12 mM spermine-HCl, 80 mM KCl, 10-60 mM $BaCl_2$ and 8-16% (v/v) MPD. Crystals were grown at 20 °C. For data collection the

crystals were cryo-protected using the corresponding well solution with 25% (v/v) ethylene glycol. Cryoprotectant was applied for approximately one minute before freezing the crystal in liquid nitrogen. Diffraction data were collected on different beamlines, including I02, I03, I04, I04-1 and I24 at Diamond Light Sources and ID23-1 and ID29 at ESRF. Data were indexed, integrated and scaled using XDS (Kabsch, 2010) or iMOSFLM and Scala from the CCP4 suite of programs (CCP4, 1994; Winn et al, 2011). Structures were solved by performing molecular replacement using PDB entry 3GX5 (Montange & Batey, 2006) or 4B5R as a preliminary model. The structures were refined using Phenix refine, and the model was built using COOT (Emsley & Cowtan, 2004). The composite omit map was calculated using Phenix (Adams et al, 2010). Crystallographic statistics for Kt-7 variants in the SAM-I riboswitch are presented in Table S6.

Sequence alignment and analysis.

Bacterial Kt-7 and Kt-46 sequences were taken from the Comparative RNA Web Site. Specific k-turn regions were aligned manually using Jalview 2.8 (Waterhouse et al, 2009). This resulted in the analysis 2,722 of Kt-7 and 3,181 of Kt-46 sequences. 4,755 SAM-I riboswitch and 9,235 U4 snRNA sequences were taken from the Rfam database (Burge et al, 2013). All sequence composition and covariation analysis was calculated using a modified version of Jalview, that was kindly provided by Dr James Procter (University of Dundee).

References

Adams PD, Afonine PV, Bunkoczi G, Chen VB, Davis IW, Echols N, Headd JJ, Hung LW, Kapral GJ, Grosse-Kunstleve RW, McCoy AJ, Moriarty NW, Oeffner R, Read RJ, Richardson DC, Richardson JS, Terwilliger TC, Zwart PH (2010) PHENIX: a comprehensive Python-based system for macromolecular structure solution. *Acta Crystallogr D Biol Crystallog* **66:** 213-221

Beaucage SL, Caruthers MH (1981) Deoxynucleoside phosphoramidites - a new class of key intermediates for deoxypolynucleotide synthesis. *Tetrahedron Letters* **22:** 1859-1862

Burge SW, Daub J, Eberhardt R, Tate J, Barquist L, Nawrocki EP, Eddy SR, Gardner PP, Bateman A (2013) Rfam 11.0: 10 years of RNA families. *Nucleic acids research* **41:** D226-232

CCP4 (1994) The CCP4 suite: programs for protein crystallography. *Acta Crystallogr D Biol Crystallogr* **50:** 760-763

Chen VB, Arendall WB, 3rd, Headd JJ, Keedy DA, Immormino RM, Kapral GJ, Murray LW, Richardson JS, Richardson DC (2010) MolProbity: all-atom structure validation for macromolecular crystallography. *Acta Cryst D* **66:** 12-21

Daldrop P, Lilley DMJ (2013) The plasticity of a structural motif in RNA: structural polymorphism of a kink turn as a function of its environment. *RNA* **19:** 357-364

Emsley P, Cowtan K (2004) Coot: model-building tools for molecular graphics. *Acta Crystallogr D Biol Crystallogr* **60:** 2126-2132

Huang L, Yin P, Zhu X, Zhang Y, Ye K (2011) Crystal structure and centromere binding of the plasmid segregation protein ParB from pCXC100. *Nucleic acids res* **39:** 2954-2968

Kabsch W (2010) XDS. *Acta Cryst D* **66:** 125-132

McCoy AJ, Grosse-Kunstleve RW, Adams PD, Winn MD, Storoni LC, Read RJ (2007) Phaser crystallographic software. *Journal of applied crystallography* **40:** 658-674

Montange RK, Batey RT (2006) Structure of the S-adenosylmethionine riboswitch regulatory mRNA element. *Nature* **441:** 1172-1175

Nagai K, Oubridge C, Jessen TH, Li J, Evans PR (1990) Crystal structure of the RNA-binding domain of the U1 small nuclear ribonuclearprotein A. *Nature* **348:** 515-520

Waterhouse AM, Procter JB, Martin DMA, Clamp M, Barton GJ (2009) Jalview Version 2 - a multiple sequence alignment editor and analysis workbench. *Bioinformatics* **25:** 1189-1191

Wilson TJ, Zhao Z-Y, Maxwell K, Kontogiannis L, Lilley DMJ (2001) Importance of specific nucleotides in the folding of the natural form of the hairpin ribozyme. *Biochemistry* **40:** 2291-2302

Winn MD, Ballard CC, Cowtan KD, Dodson EJ, Emsley P, Evans PR, Keegan RM, Krissinel EB, Leslie AG, McCoy A, McNicholas SJ, Murshudov GN, Pannu NS, Potterton EA, Powell HR, Read RJ, Vagin A, Wilson KS (2011) Overview of the CCP4 suite and current developments. *Acta Cryst D* **67:** 235-242
