# Supplementary material for: A critical base pair in k-turns determines the conformational class adopted, and correlates with biological function
Source: Nucleic Acids Res. 2016 Mar 25;44(11):5390–8. doi: 10.1093/nar/gkw201 (PMC4914095; doi:10.1093/nar/gkw201)
Supplement: SUPPLEMENTARY DATA [file supp_44_11_5390__index.html]

A critical base pair in k-turns determines the conformational class adopted, and correlates with biological function — A critical base pair in k-turns determines the conformational class adopted, and correlates with biological function — SUPPLEMENTARY DATA 

# A critical base pair in k-turns determines the conformational class adopted, and correlates with biological function

## SUPPLEMENTARY DATA

- SUPPLEMENTARY DATA
